# Association of depressive symptoms and risk of knee pain: the moderating effect of sex

**DOI:** 10.1186/s12891-021-04511-2

**Published:** 2021-07-26

**Authors:** Haiyan Hu, Wenjun Liu, Yang Liu, Jay Pan, Xiaozuo Zheng

**Affiliations:** 1 HEOA Group, West China School of Public Health and West China Fourth Hospital, Sichuan University, No. 16, Section 3, Ren Min Nan Road, Chengdu, 610041 China; 2 Department of Spine Surgery, The First College of Clinical Medical Science, China Three Gorges University, Yichang, 443003 Hubei China; 3 Department of Spine Surgery, Yichang Central People’s Hospital, Yichang, 443003 Hubei China; 4 Institute for Healthy Cities and West China Research Center for Rural Health Development, Sichuan University, Chengdu, 610041 Sichuan China; 5 Department of Orthopaedic Surgery, Third Hospital of Hebei Medical University, No.139 Ziqiang Road, Shijiazhuang, 050051 Hebei China

**Keywords:** Knee pain, Musculoskeletal pain, Chronic pain, Depressive symptoms, Mental disorder, Cohort study, Moderation analysis, Moderating effect, Sex

## Abstract

**Background:**

Depression has been shown in some studies to be associated with knee pain. Females were widely recognized as more vulnerable to depression and knee pain than males. However, the role of sex in this correlation was under-researched. This study aimed to investigate the association between depressive symptoms and subsequent knee pain, as well as whether and how sex would moderate this association based on a four-wave (Wave 1 in 2010–2011, Wave 2 in 2013, Wave 3 in 2015, and Wave 4 in 2018) longitudinal study among middle-aged and elderly Chinese adults.

**Methods:**

Seventeen thousand seven hundred eight participants were recruited and followed in the China Health and Retirement Longitudinal Study (CHARLS). Ten thousand four hundred fifty-one entered the final analysis based on the inclusion and exclusion criteria. Knee pain was assessed by self-report. Depressive symptoms were evaluated using the validated 10-item Center for Epidemiological Studies-Depression Scale (CESD-10). Cox proportional hazards models were used to calculate hazard ratios with 95% confidence intervals (CIs) after controlling potential confounders to examine the association between depressive symptoms and subsequent incident and persistent knee pain. Non-linear association of depressive symptoms score (CESD-10) and risk of knee pain was also investigated via applying 3-knotted restricted cubic spline regression. An interaction term of depressive symptoms status and sex was added to investigate the moderating effect of sex on the relationship between depressive symptoms status and the risk of knee pain.

**Results:**

The median follow-up time was seven years for all the outcomes. Participants with depressive symptoms were 1.45 times (95% CI: 1.34–1.56) and 2.16 times (95% CI: 1.85–2.52) more likely to develop the incident and persistent knee pain after multivariable were adjusted, compared with those without depressive symptoms. There was a non-linear association between CESD-10 score and risk of knee pain. Compared with females, males had an enhanced correlation between depressive symptoms status and knee pain (multivariable-adjusted HR: 1.22, 95% CI: 1.05–1.42 and HR: 1.57, 95% CI: 1.14–2.17 for the incident and persistent knee pain, respectively).

**Conclusion:**

Depressive symptoms are independently associated with an excess risk of knee pain, with a stronger correlation for males than females among middle-aged and elderly Chinese adults.

**Supplementary Information:**

The online version contains supplementary material available at 10.1186/s12891-021-04511-2.

## Plain English Summary

This study investigated the incidence of knee pain, the correlation of depressive symptoms with knee pain, and the moderating effect of sex on this correlation in a nationally representative cohort of middle-aged and elderly Chinese adults. Results suggested that depressive symptoms were associated with an excess risk of knee pain, with a stronger correlation for males than females.

**Supplementary Information:**

The online version contains supplementary material available at 10.1186/s12891-021-04511-2.

## Highlights

- Depressive symptoms significantly increase the risk of knee pain.
- Females are more vulnerable to depression and knee pain than males.
- The correlation between depressive symptoms and knee pain is more significant in males than in females.

## Introduction

Knee pain, one of the most prevalent musculoskeletal (MSK) pain disorders, affects large proportions of the population [1]. It was estimated that approximately 33% of middle-aged and elderly adults were affected by painful knees [2, 3]. Its prevalence has increased by almost 65% over the past two decades [1] and is expected to continue as life expectancies grow. Chronic knee pain is commonly defined as knee pain that persists longer than three months or beyond the normal tissue healing duration [4]. It is a leading cause of disability, one of the main reasons for medical treatment-seeking behavior in primary care settings, and contributes to a substantial socioeconomic burden [5]. However, the incidence of knee pain among the general population remains under-researched. Identifying the significant risk factors for knee pain is valuable for establishing prevention plans for the people at risk and providing clinical evidence to develop more effective management strategies for knee pain sufferers from progressing into chronic complaints.

In the most widely referenced biopsychosocial framework, both intra-articular and extra-articular risk factors may lead to knee pain [5–7]. Psychological factors are frequently recognized as correlated with knee pain [5, 8], with depression being recognized as one of the most common and disease-laden psychological disorders, particularly for the middle-aged and elderly population. For the 50–74 age group, the percentage change in the number of disability-adjusted life-years (DALYs) attributing to depression has risen by 107.3% over the past three decades [9]. Previous studies have suggested that an increase in inflammatory cytokines and altered neurotransmitter levels associated with depression contributes to changing physical pain perception threshold, while the underline mechanism was not fully understood [10–12]. Therefore, depression may aggravate the perception of knee pain.

Depression has been shown in some studies to be associated with knee pain [13–22]. Some detected a significant association [16–20, 22], while others failed to [13–15, 21]. Inherent heterogeneity including, but not limited to, study design, sample size, sample selection, and statistical modeling method may have contributed to this variation in the observed depression-knee pain correlation. Besides, whether and how sex moderated the relationship between depression and knee pain was not well-characterized. Although females have been widely perceived as more vulnerable to depression [23, 24] and pain disorders [25, 26], sex is often examined as an averaged effect in previous studies [16, 17, 19, 20]. More specific investigation into the role of sex in the depression-knee pain relationship would improve knee pain prevention, care, and management for both sexes.

This study aimed to 1) investigate the incidence rate of knee pain among middle-aged and elderly adults according to the status of the depressive symptoms, 2) examine the correlation of depressive symptoms and subsequent knee pain, and 3) explore the moderating role of sex on this relationship.

## Methods

### Study design

Data used in this study were obtained from the China Health and Retirement Longitudinal Study (CHARLS), available to the public at http://charls.pku.edu.cn/index/en.html after registration and application. CHARLS was an ongoing nationally representative population-based cohort survey in China, harmonized with the US Health and Retirement Study (HRS) and related surveys on aging worldwide [27]. Zhao et al.’ described details for the design and performance of CHARLS [28]. The Biomedical Ethics Review Committee of Peking University approved the CHARLS study (No: IRB00001052-11,015) [28]. Ethics approval for the use of CHARLS data was obtained from the University of Newcastle Human Research Ethics Committee (H-2015–0290).

### Participants and data collection

Seventeen thousand seven hundred eight participants from 28 provinces, municipal cities, and autonomous regions were recruited through the multistage probability-proportional-to-size (PPS) sampling strategy in the baseline survey (Wave 1, between June 2011 and March 2012). Follow-ups were performed in Wave 2 (2013), Wave 3 (2015), and Wave 4 (2018) [27]. Participants reflect the middle-aged and elderly Chinese population collectively [27]. Respondents were interviewed by trained staff using face-to-face laptop-assisted questionnaires [28]. The response rate for the baseline survey (Wave 1) was 80.5%. CHARLS interviewers obtained informed consent from all participants before the survey [27].

### Measures

#### Knee pain

The study outcome was an incident of knee pain. In the questionnaires, knee pain was assessed by asking whether the participants had experienced knee pain in the past month. Following previous studies [29, 30], participants were classified into two subgroups: persistent and incident knee pain. Participants with persistent knee pain refer to those reporting knee pain during two or more consecutive follow-up waves. Participants with incident knee pain refer to those subjects with pain in one or more non-consecutive waves.

#### Depressive symptoms

Depressive symptoms were evaluated using the validated Center for Epidemiological Studies-Depression Scale (CESD-10), which rates symptoms related to depression [31, 32]. Ten items’ summation score may range from 0 to 30, with higher values indicating more severe depressive symptoms. A total CESD-10 score of 10 or greater was commonly used to indicate clinically significant depression for Chinese adults [29].

#### Covariates

Consistent with previous studies [17, 29, 33], the following covariables were included. The interviewer-administered questionnaire obtained all the covariates at the baseline survey.

- Demographic characteristics included age (continuous: years) and sex (binary: male or female). In the moderation analysis, sex was considered as the moderator.
- Socioeconomic status (SES) included education attainment (ordinal: illiterate, primary school, middle school, high school or above), marital status (binary: currently married or partnered, or single), neighborhood (binary: rural or urban), household income per capita (ordinal: categorized into four quartiles [Qs], from Q1[the lowest] to Q4 [the highest]), occupation (binary: non-farming or farming).
- Health status included the number of common chronic comorbidities, body mass index (BMI) level, and injury history (binary: with or without). The number of common chronic comorbidities (ordinal: none, one, two, three, or above). Chronic diseases involved hypertension, diabetes, dyslipidemia, chronic lung diseases, liver diseases, heart disease, stroke, cancer, chronic kidney diseases, digestive diseases, etc. Following the Working Group on Obesity in China, body mass indexes (BMI) were categorized into four levels (ordinal: underweight [BMI < 18.5 kg/m^2^], normal [18.5 ≤ BMI < 24 kg/m^2^], overweight [24 ≤ BMI < 28 kg/m^2^], and obesity [BMI ≥ 28 kg/m^2^] [34]. Injury history was assessed by asking the respondents “Have you ever been in a traffic accident or any other kind of major accidental injury and received medical treatment?”. If the respondent said yes to the question, the respondent was classified as having injury history.
- Health-related behavior included cigarette smoking (binary: yes or no) and drinking (ordinal: none, less than once a month or more than once a month).

## Research samples

**Fig. 1:**
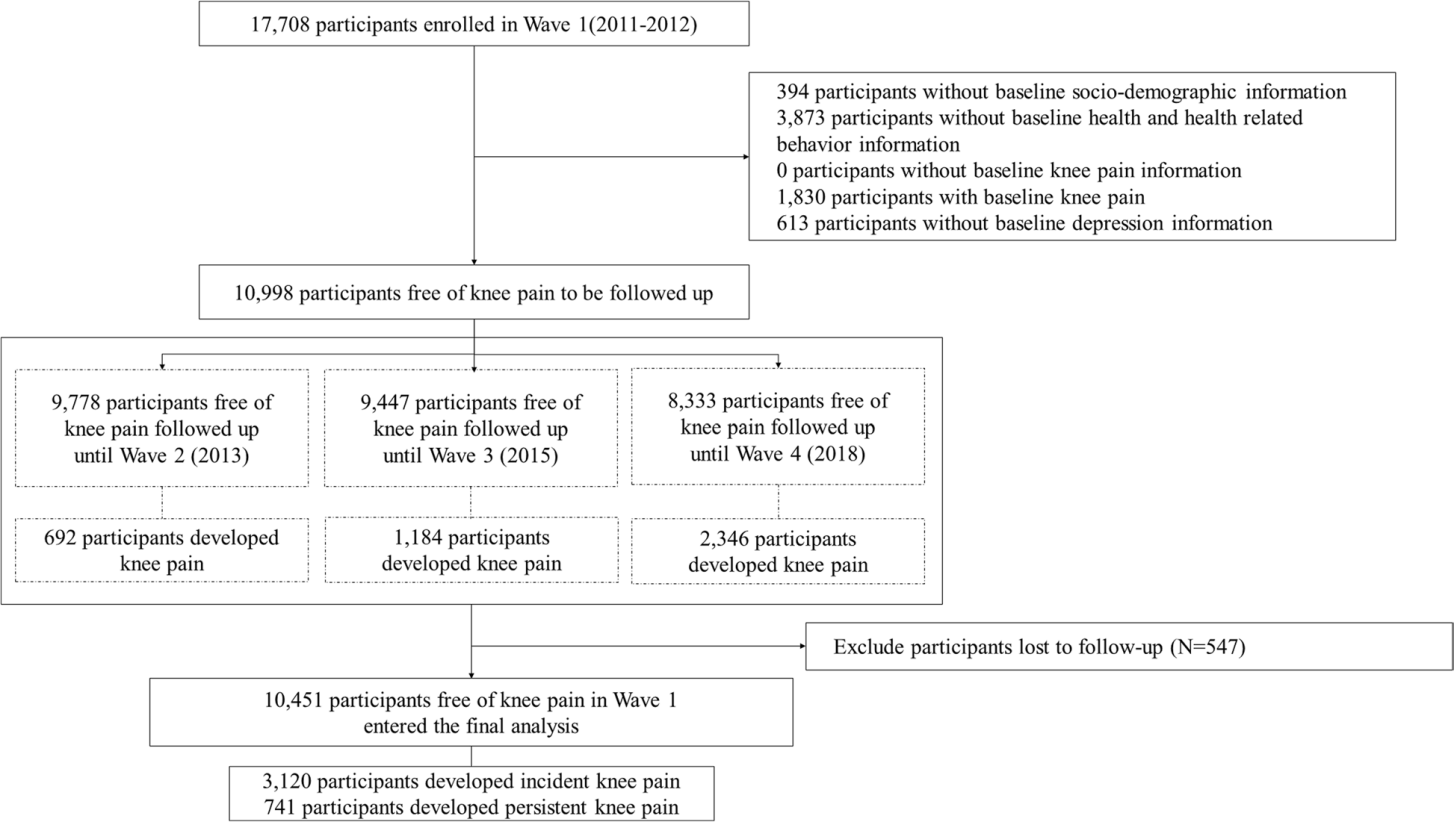
Flow diagram of participants for examining the association of depressive symptoms status with risk of knee pain

For this analysis, we selected participants who were followed up for at least one wave since the baseline. We excluded participants without baseline information for socio-demographics, health and health-related behavior, knee pain or depressive symptoms, and those reporting baseline knee pain. Figure 1 presents the flow chart of screening the research population for this study.

### Statistical analysis

Data were described as means with standard deviations (SDs) for quantitative variables and frequency with proportions for binary and ordinal variables. Baseline characteristics were summarized according to the presence of depressive symptoms. Between-group comparisons were conducted according to depressive symptoms using the Student’s T-test, Chi-square test, and the Wilcoxon rank-sum test, as appropriate.

The person-year of follow-up for each respondent was calculated from the baseline wave (2011–2012) to the years of confirming knee pain, fail to follow-up, or the end of follow-up, whichever came first. The incidence rates per 1,000 person-years and 95% confidence intervals (CIs) of the incident and persistent knee pain were calculated as a whole and by baseline depression status.

To examine the association between depressive symptoms and subsequent knee pain, Cox proportional hazards models were used to calculate hazard ratios (HRs) with 95% CIs. We reported both the unadjusted model (crude model) and the covariables-adjusted model (adjusted model) in the main text. Schoenfeld residuals were tested for evaluating the proportional hazards assumption for Cox proportional hazards regression, using the “estat phtest” command in Stata software. A *p*-value larger than 0.05 indicates that there is no evidence that the proportional-hazards assumption has been violated. Only regression models satisfying this assumption were reported in the text. Besides, we explored the potential non-linear associations of depressive symptoms score (CES-D) with the incident and persistent knee pain in the adjusted model applying 3-knotted restricted cubic spline regression through the Stata command “mkspline”. A *p*-value of less than 0.05 for test linearity hypotheses after estimation indicates that the null hypothesis would be rejected, and there would be a non-linear association.

26.56% (4,368 of 16,447) of total observations containing missing values were discarded from the initial analysis, known as listwise deletion or complete case analysis. To account for this method's significant drawback, this study conducted multiple imputations of chained equations approach based on the baseline characteristics, followed by Cox proportional hazards regression modeling. Under the assumption that these missing values are missing at random (MAR), a simulation-based three-step imputation procedure was conducted. First, missing values were replaced with multiple sets of imputed values from the regress, logit, or ordinal logit model as appropriate to form fifty completed datasets. Second, standard complete data analyses were performed based on each dataset imputed from step 1. Third, results from step 2 were consolidated into multiple imputation inferences using the Stata command “mi estimate” [35].

All statistical analyses were performed in Stata 16 (StataCorp LP, College Station, Texas), and the significance was set at a two-tailed $\alpha$ of less than 0.05.

## Results

### Participants’ characteristics

Table 1 presents the participants’ baseline characteristics. A total of 10,451 participants entered the final analysis, of which 3,368 (32.23%) were depressed at baseline. The mean age was 59.45 years (SD: 9.58 years). Participants were mostly female (*N* = 5,349, 51.18%), educated less than six years (illiterate: *N* = 2,653, 25.39%; primary school: *N* = 4,239, 40.56%), currently married or partnered (*N* = 9,294, 88.93%), living in rural areas (*N* = 8,361, 80.00%), normal weighted (*N* = 5,551, 53.11%), non-smokers (*N* = 7,174, 68.64%), non-drinkers (*N* = 6,950, 66.50%), without injury history (*N* = 10,022, 95.90%) and suffered from one or more chronic diseases (*N* = 6,694, 64.05%). Group comparison results showed that depressive symptoms were more prevalent in the senior, female, less educated, divorced, widowed or never married, non-smokers, non-drinkers, and those living in rural areas, economically disadvantaged, engaged in agriculture, with injury history, and suffering from chronic diseases (*p* < 0.001).

**Table 1:** Baseline characteristic of study participants according to depressive symptoms status (*N* = 10,451)

	Depressive symptoms		Total (*N* = 10,451)	*p* value
	With (*N* = 3,368)	Without (*N* = 7,083)		
Age, mean (SD), years	60.19(9.72)	59.10(9.49)	59.45(9.58)	< 0.001
Sex, n (%)				< 0.001
Female	2,020(59.98)	3,329(47.00)	5,349(51.18)	
Male	1,348(40.02)	3,754(53.00)	5,102(48.82)	
Education attainment, n (%)				< 0.001
Illiterate	1,075(31.92)	1,578(22.28)	2,653(25.39)	
Primary school	1,483(44.03)	2,756(38.91)	4,239(40.56)	
Middle school	600(17.81)	1,708(24.11)	2,308(22.08)	
High school or above	210(6.24)	1,041(14.70)	1,251(11.97)	
Marital status, n (%)				< 0.001
Otherwise (divorced, widowed, never married)	512(15.20)	645(9.11)	1,157(11.07)	
Currently married or partnered	2,856(84.80)	6,438(90.89)	9,294(88.93)	
Type of neighborhood, n (%)				< 0.001
Rural	2,876(85.39)	5,485(77.44)	8,361(80.00)	
Urban	492(14.61)	1,598(22.56)	2,090(20.00)	
Household income per capita, n (%)				< 0.001
Quantile 1 (lowest)	1,088(32.30)	1,524(21.52)	2,612(24.99)	
Quantile 2	945(28.06)	1,668(23.55)	2,613(25.00)	
Quantile 3	770(22.86)	1,843(26.02)	2,613(25.00)	
Quantile 4 (highest)	565(16.78)	2,048(28.91)	2,613(25.00)	
Occupation, n (%)				< 0.001
Non-farming	1,310(38.90)	3,024(42.69)	4,334(41.47)	
Farming	2,058(61.10)	4,059(57.31)	6,117(58.53)	
Body mass index (BMI) level, n (%)				< 0.001
Underweight (BMI < 18.5)	286(8.49)	354(5.00)	640(6.12)	
Normal (18.5 ≤ BMI < 24)	1,885(55.97)	3,666(51.76)	5,551(53.11)	
Overweight (24 ≤ BMI < 28)	888(26.37)	2,189(30.90)	3,077(29.44)	
Obesity (BMI ≥ 28)	309(9.17)	874(12.34)	1,183(11.32)	
Cigarette smoking, n (%)				< 0.001
No	2,417(71.76)	4,757(67.16)	7,174(68.64)	
Yes	951(28.24)	2,326(32.84)	3,277(31.36)	
Drinking, n (%)				< 0.001
None	2,439(72.42)	4,511(63.69)	6,950(66.50)	
Less than once a month	232(6.89)	599(8.46)	831(7.95)	
More than once a month	697(20.69)	1,973(27.86)	2,670(25.55)	
Injury, n (%)				< 0.001
Without	3,163(93.91)	6,859(96.84)	10,022(95.90)	
With	205(6.09)	224(3.16)	429(4.10)	
Number of chronic diseases, n (%)				< 0.001
None	878(26.07)	2,879(40.65)	3,757(35.95)	
One	1,046(31.06)	2,254(31.82)	3,300(31.58)	
Two	727(21.59)	1,181(16.67)	1,908(18.26)	
Three or above	717(21.29)	769(10.86)	1,486(14.22)	

*Notes*: *SD* standard deviation

### Incidence rate and hazard ratio of knee pain according to depressive symptoms status

Ten thousand four hundred fifty-one respondents were followed up (median: 7 years). A total of 3,120 developed incident knee pain, with the IR being 46.44, 65.13, and 37.66 per 1000 person-years for all the respondents, respondents with and without depressive symptoms, respectively (Table 2, panel A). A total of 741 developed persistent knee pain, with the IR being 14.5, 28.51, and 8.69 per 1000 person-years for all the respondents, respondents with and without depressive symptoms, respectively (Table 2, panel B).

**Table 2:** Incidence rate and hazard ratio of knee pain according to depressive symptoms status

**Outcome**	**Case / person-years**	**Incidence rate, per 1000 person-years**	**HR (95% CI)**	
			**Crude model**	**Adjusted model**
**Panel A: Incident knee pain**				
Depressive symptoms				
Without	1721/45701	37.66	1 [Reference]	1 [Reference]
With	1399/21480	65.13	1.75(1.63–1.88)	1.45(1.34–1.56)
Total	3120/67181	46.44		
**Panel B: Persistent knee pain**				
Depressive symptoms				
Without	314/36125	8.69	1 [Reference]	1 [Reference]
With	427/14977	28.51	3.33(2.88–3.85)	2.16(1.85–2.52)
Total	741/51102	14.50		

*Notes*: Depressive symptoms were defined as a score of 10 or greater on the 10-item Center for Epidemiologic Studies Depression Scale

Participants with depressive symptoms were consistently at higher risk of developing incident knee pain (crude HR: 1.75, 95% CI [1.63–1.88]; adjusted HR: 1.45, 95% CI [1.34–1.56], Table 2 panel A) and persistent knee pain (crude HR: 3.33, 95% CI [2.88–3.85]; adjusted HR: 2.16, 95% CI [1.85–2.52], Table 2 panel B). Multiple imputations inference of depression’s contribution did not show appreciable changes for incident knee pain (adjusted HR: 1.39, 95% CI [1.32–1.48]) or persistent knee pain (adjusted HR:2.09, 95% CI [1.86–2.34]).

### Non-linear association of depressive symptoms scores with knee pain

**Fig. 2:**
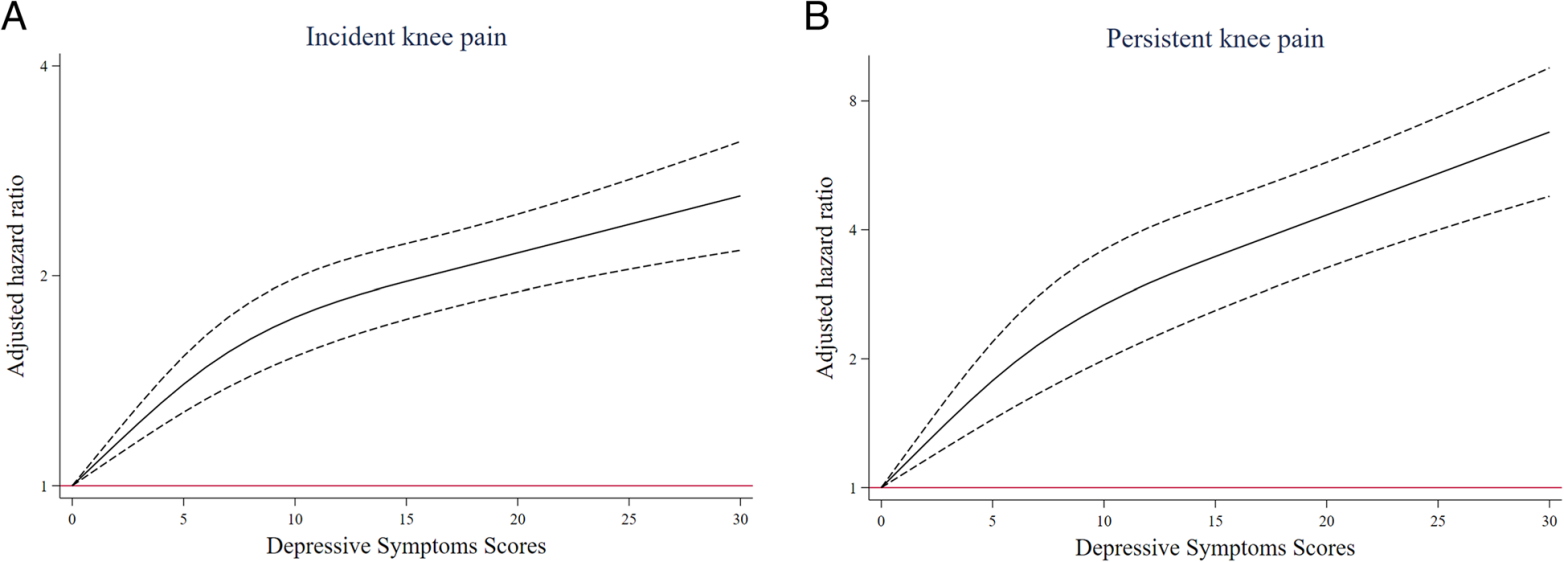
Adjusted hazard ratios (HRs) of knee pain according to depressive symptoms scores. Notes: Graphs show HRs for incident knee pain (Panel A) and persistent knee pain (Panel B). HRs were adjusted for demographic characteristics, socioeconomic status (SES), health status, and health-related behavior. Data were fitted by a 3-knots restricted cubic spline Cox proportional hazards regression model. The depressive symptoms score ranges from 0 to 30, with the highest score representing the highest risk of depressive symptoms. Solid lines indicate HRs, and dashed lines indicate 95% CIs

A non-linear and positive association between the CES-D total score and risk of knee pain using restricted cubic spline regression was found (for linearity, Chi-square of Wald tests = 26.30, *p* < 0.001 for incident knee pain; Chi-square of Wald tests = 14.31, *p* < 0.001 for persistent knee pain (Fig. 2).

### Moderating effect of sex on the relationship between depressive symptoms status and knee pain

Significant HRs greater than 1.00 were observed for interactions of depressive symptoms and sex. These results indicated that the correlation of depressive symptoms with subsequent incident knee and persistent knee pain was 1.22 (95% CI: 1.05–1.42) and 1.57 (95% CI: 1.14–2.17) times stronger for males compared with females, respectively (Table 3, panel A and B). Results of moderating effect analyses were robust when multiple imputations were performed for incident knee pain (adjusted HR: 1.21, 95% CI [1.08–1.36]) and persistent knee pain (adjusted HR: 1.48, 95% CI [1.16–1.89]).

**Table 3:** Moderating effect of sex on the relationship between depressive symptoms status and the risk of knee pain

Outcome	Hazard ratio (95% CI)
**Panel A: Incident knee pain**	
Depressive symptoms (Ref = without)	1.35(1.23–1.48)
Sex (Ref = female)	0.66(0.58–0.74)
Interaction (Depressive symptoms × Sex)	1.22(1.05–1.42)
**Panel B: Persistent knee pain**	
Depressive symptoms (Ref = without)	1.88(1.57–2.26)
Sex (Ref = female)	0.37(0.28–0.49)
Interaction (Depressive symptoms × Sex)	1.57(1.14–2.17)

*Notes*: Depressive symptoms were defined as a score of 10 or greater on the 10-item Center for Epidemiologic Studies Depression Scale

## Discussion

The main findings were three-fold. First, incidence rates of knee pain in participants with depressive symptoms were consistently higher than those without depressive symptoms. Second, depressive symptoms were independently associated with the development of knee pain after controlling potential confounders. Third, sex moderated this association where depressed males were at a higher risk of developing future knee pain than depressed females.

### Depressive symptoms increased the risk of knee pain

Depressive symptoms were associated with an approximately 1.45-to-2.16-fold higher risk of knee pain after adjustment for confounding factors. This result was comparable with previous findings that depressive symptoms were correlated with an increased risk of knee pain. Wright et al. [16] reported a statistically significant simple correlation coefficient between depressive symptoms and knee pain among 275 knee OA recruited from the urban community. Nevertheless, the sample inclusion limited the generalizability of the results to the general population due to the exclusion of participants with moderate to high levels of physical activity at baseline [16]. Peat and Thomas [18] performed a nested case-control study, which included 57 cases and 228 controls aged over 50 years. They reported that an increase in depressive symptoms was associated with substantial deterioration of knee pain. Riddle et al. [22] found depressive symptoms predictive for worsening self-reported knee pain with a small magnitude effect, indicating depression was required to persist for a considerable year to produce an appreciable impact on future knee pain.

Although multiple studies have investigated the relationship between depressive symptoms and knee pain, the degree to which depressive symptoms are associated with the risk of future knee pain among the most vulnerable middle-aged and elderly general population remains unknown. Most of the current studies were cross-sectional studies [13–17, 20, 21], based on which the temporal direction of such relationships is difficult to discern; some previous studies examined osteoarthritis (OA) patients recruited in the clinics or hospitals [14, 15]. Therefore, findings from these studies may not be generalizable to the general population. Knee pain is usually under-reported, under-diagnosed, and under-treated, particularly among the community-dwelling middle-aged and older adults [36]. Papandony et al. systematically reviewed patients' perceived health service needs for OA care. They found that increases in frequency and intensity of symptoms and disturbance to their daily life activities may motivate their healthcare-seeking behaviors [37]. It may lead to differences in knee pain intensity, frequency, or interference among participants recruited from medical institutions and community settings. Previous studies have also suggested that the association of depressive symptoms and pain would differ by pain characteristics [38]. Therefore, this association might vary among different research populations. The link found in studies focusing on OA patients recruited in the clinics or hospitals could not apply to the general population. The research on the general population may be of more relevance to public health and preventive care. It is particularly significant in light of the prevalence and impact of comorbid depression and knee pain. Specifying this association among the general population would help identify the population at risk and promote primary prevention strategies and secondary care management that may prevent incident knee pain from progressing into chronicity.

The relationship between depressive symptoms and risk of knee pain persisted when the subjects were stratified by the phenotype of knee pain. Specifically, the correlation between depressive symptoms and persistent knee pain (HR: 2.16) was much stronger than that of depressive symptoms and incident knee pain (HR: 1.45). It indicated that the association of depressive symptoms with future knee pain was more evident for chronicity than non-chronicity.

These findings were supported by previous functional studies, which revealed that the emotional processing within the insula in depressed patients was shifted topologically toward the insula areas, which were generally involved in pain processing [39, 40]. Overlapping between emotional and pain-processing circuitry supported the association between depressive symptoms and pain experience [40]. The changes could last long and thus produce a persistent effect on pain symptoms [41].

Central sensitization could be another explanation for this association [42]. Central sensitization could be manifested clinically as hyperalgesia (exaggerated and prolonged in response to noxious stimuli), secondary allodynia (painful sensation to a normally non-painful stimulus), expansion of the receptive field (pain that extends beyond the area of peripheral nerve supply), and unusually prolonged pain after the stimulus has been removed (usually burning, throbbing, tingling, or numbness) [43]. Therefore, chronic or persistent pain was considered as being maintained in part by central sensitization [44]. However, central sensitization could be driven by neuroinflammation, one of the common pathophysiology for depression and pain [44–47]. Neuroinflammation could be a joint driving force for chronic pain and neuropsychiatric diseases such as depression [44]. This study revealed a significant correlation between depressive symptoms and persistent knee pain. Future researchers would further explore other aspects of central sensitization, such as the relationship between depression and multi-site or widespread pain.

### Sex moderated the association of depressive symptoms and the risk of knee pain

Analysis of moderating variables revealed variations in the magnitude of the depression-knee pain relationship, not the direction of the depression difference. That is, all HRs for the risk of knee pain were more significant than 1.0 among different sexes. These findings indicated that depressive symptoms contributed to future knee pain for both sexes, emphasizing the consistency that depressed respondents had an elevated risk for knee pain than those non-depressed. However, compared with females, males had an enhanced correlation between depression and knee pain (1.22–1.57 times). These results were consistent for different knee pain phenotypes, with the most substantial moderation effect observed for the persistent knee pain (1.57 times).

Evidence suggested that sex differences in the depressive symptoms-pain association may be caused by multiple biological-social-psychological mechanisms, including sex hormones and their interaction with the immune system, gender roles, pain coping, and catastrophizing [48, 49]. The sex difference in depressive symptoms may also be caused by biological factors, psychological factors, and multiple factors at micro and macro levels [50].

Nevertheless, sex was often controlled as a confounder in the analytic models in previous studies [51]. To date, research on sex differences in the relationship between depressive symptoms and future knee pain was relatively lacking. Perruccio et al. [51] examined the influence of depressive symptoms on post-total knee arthroplasty (TKA) pain among TKA patients. They found that sex moderated the effect of depressive symptoms on post-surgery knee pain, with a more significant effect being observed for males than for females [51]. Our findings were comparable with Perruccio et al.’. However, our study had a more generalized population, a larger sample size, and a longer follow-up.

The differential correlation strength of depressive symptoms with knee pain between varied sexes, which is more significant for males, did not suggest that depressive symptoms were inconsequential in females. Accumulated evidence indicated that females were at substantially greater risk for depressive symptoms and knee pain [52, 53]. Males may be less likely to suffer from depressive symptoms and painful knees than females; however, this does not mean that depressed males would be less impaired by this correlation. Conservatively speaking, our findings could be interpreted as equal attention should be paid to the males, who were in a disadvantaged position in the relationship between depressive symptoms and risk of knee pain. It is very important to avoid overlooking depressive symptoms among males and overemphasizing depressive symptoms among females at the clinical and primary care settings to eliminate or minimize the harm of sex stereotypes, in other words, to refrain from over-diagnosis and over-treatment of females, and under-diagnosis and under-reporting of males [53]. Furthermore, the correlation of depressive symptoms with subsequent knee pain cannot be assumed to be the same for different sexes, which may mask or weaken the underlying association for males [51].

### Strengths and potential limitations

This study used a longitudinal research design, adopted a large sample size from community-based rather than general clinics or hospital-based populations, included a significant number of participants who reported symptoms but may not have sought medical help, thereby increasing the generalizability of our findings. However, this study was not without limitation. First, there were 26.56% of participants ineligible due to missing data on interest variables. Second, measurement errors may perhaps not be entirely excluded and would be another source of bias. Although CESD-10 used to evaluate depression has been validated and shown fair reliability among Chinese community-dwelling adults [29], misclassification of depressive symptoms would also exist due to the unavailability of a clinical diagnostic tool for depression in CHARLS. Knee pain was measured via single self-report through binary options, i.e., yes or no. However, it may be prone to underestimate pain particularly for the older adults who would consider pain to be part of normal age-related physical declines [54]. Third, depressive symptoms would not be fixed. Instead, they could be time-variant, making assessing causality more challenging [55]. The potential reciprocal relationship between depressive symptoms and knee pain should also be noted [19, 56]. Fourth, our findings were likely to suffer from omitted variable bias that would be caused by unmeasured confounders responsible for the correlation. Therefore, though the longitudinal design was applied, the observational nature of this study did not warrant causal inferences. Our findings should be interpreted in light of, but not restricted to, the above limitations.

### Theoretical and practical implications

Despite these limitations, the findings as a whole have provided valuable information that may have implications for future practice and research. First, this study strengthened the evidence for the correlation of depressive symptoms with knee pain, particularly the chronicity of knee pain complaints among the general middle-aged and elderly population. It would be helpful to develop and provide psychological preventive and treatment programs. As the population ages, the direct and indirect costs associated with knee pain may also shift upwards, highlighting the importance of identifying those at higher risk, thereby promoting primary prevention strategies and secondary care management that may prevent incident knee pain from progressing into chronicity.

Second, the findings suggest the association of depressive symptoms with knee pain risk would be different by sexes. Therefore, it would be necessary to notice the sex-moderated association of depressive symptoms with knee pain and minimize the harm of sex stereotypes. Though males were less likely to suffer from knee pain or depressive symptoms than females, depressed males had a higher risk of knee pain than depressed females. Therefore, it is crucial to give males an adequate psychological evaluation. It is also imperative to advocate a more personalized approach to shared public health and prevention decisions in rural and urban areas, considering local cultures and resources to achieve better healthcare results from a group perspective.

Third, psychological assessments and interventions should be more accessible in the primary care settings closest to the people at risk. More efforts are needed in public education of psychological services, mental health, well-being, and local resources for promoting healthy living.

Finally, for future research, an essential next step is to design and evaluate the potential beneficial effects of preventive strategies for those at risk of knee pain and continue to improve the administration of prevention efforts. Further research is needed to compare with our findings and explain why these sex differences exist. In addition, it should be taken into account that the complexity of accurate pain assessment strategies caused by aging-related physiological changes when using knee pain assessment tools in future studies [54].

## Conclusion

This study found a positive correlation between depressive symptoms and the risk of knee pain among middle-aged and elderly Chinese adults. This correlation was moderated by sex, with a stronger association among males than females.

## Supplementary Information

**Additional file 1: Appendix Table 1.** Hazard ratio (HR) of knee pain according to depressive symptoms status after multiple imputation

**Additional file 2: Appendix Table 2.** Moderated effect of sex on the association of depressive symptoms status and knee pain

**Additional file 3.** Association between depressive symptoms and risk of incident knee pain

**Additional file 4.** Association between depressive symptoms and risk of persistent knee pain

## Data Availability

The datasets used and/or analyzed during the current study are available from the corresponding author on reasonable request.

## Abbreviations

MSK: Musculoskeletal
DALYs: Disability-adjusted life-years
CHARLS: China Health and Retirement Longitudinal Study
CESD: Center for Epidemiological Studies-Depression Scale
SES: Socioeconomic status
BMI: Body mass index
SD: Standard deviation
CI: Confidence interval
HR: Hazard ratio
MAR: Missing at random
OA: Osteoarthritis
TKA: Total knee arthroplasty
