# Supplementary material for: Association of depressive symptoms and risk of knee pain: the moderating effect of sex
Source: BMC Musculoskelet Disord. 2021 Jul 26;22:640. doi: 10.1186/s12891-021-04511-2 (PMC8314447; doi:10.1186/s12891-021-04511-2)
Supplement: Supplementary file 1 — Additional file 1: Appendix Table 1. Hazard ratio (HR) of knee pain according to depressive symptoms status after multiple imputation [file 12891_2021_4511_MOESM1_ESM.docx]

**Appendix Table 1** Hazard ratio (HR) of knee pain according to depressive symptoms status after multiple imputation

| **Outcome** | **HR** |
| --- | --- |
|  | **Adjusted model** |
| **Panel A: Incident knee pain** | |
| Depressive symptoms |  |
| Without | 1 [Reference] |
| With | 1.39(1.32-1.48) |
| **Panel B: Persistent knee pain** | |
| Depressive symptoms |  |
| Without | 1 [Reference] |
| With | 2.09(1.86-2.34) |

*Notes: Adjusted model. CI Confidence Interval*
