# Supplementary material for: Association of depressive symptoms and risk of knee pain: the moderating effect of sex
Source: BMC Musculoskelet Disord. 2021 Jul 26;22:640. doi: 10.1186/s12891-021-04511-2 (PMC8314447; doi:10.1186/s12891-021-04511-2)
Supplement: Supplementary file 2 — Additional file 2: Appendix Table 2. Moderated effect of sex on the association of depressive symptoms status and knee pain [file 12891_2021_4511_MOESM2_ESM.docx]

**Appendix Table 2** Moderated effect of sex on the association of depressive symptoms status and knee pain

| **Outcome** | **Hazard ratio (95% CI)** |
| --- | --- |
| **Panel A: Incident knee pain** |  |
| Depressive symptoms (Ref=without) | 1.31(1.22-1.40) |
| Sex (Ref=female) | 0.66(0.60-0.73) |
| Interaction (Depressive symptoms × Sex) | 1.21(1.08-1.36) |
| **Panel B: Persistent knee pain** |  |
| Depressive symptoms (Ref=without) | 1.85(1.62-2.12) |
| Sex (Ref=female) | 0.41(0.33-0.51) |
| Interaction (Depressive symptoms × Sex) | 1.48(1.16-1.89) |

*Notes: Adjusted model. CI Confidence Interval*
