# Supplementary figures and images for: Association of depressive symptoms and risk of knee pain: the moderating effect of sex
Source: BMC Musculoskelet Disord. 2021 Jul 26;22:640. doi: 10.1186/s12891-021-04511-2 (PMC8314447; doi:10.1186/s12891-021-04511-2)

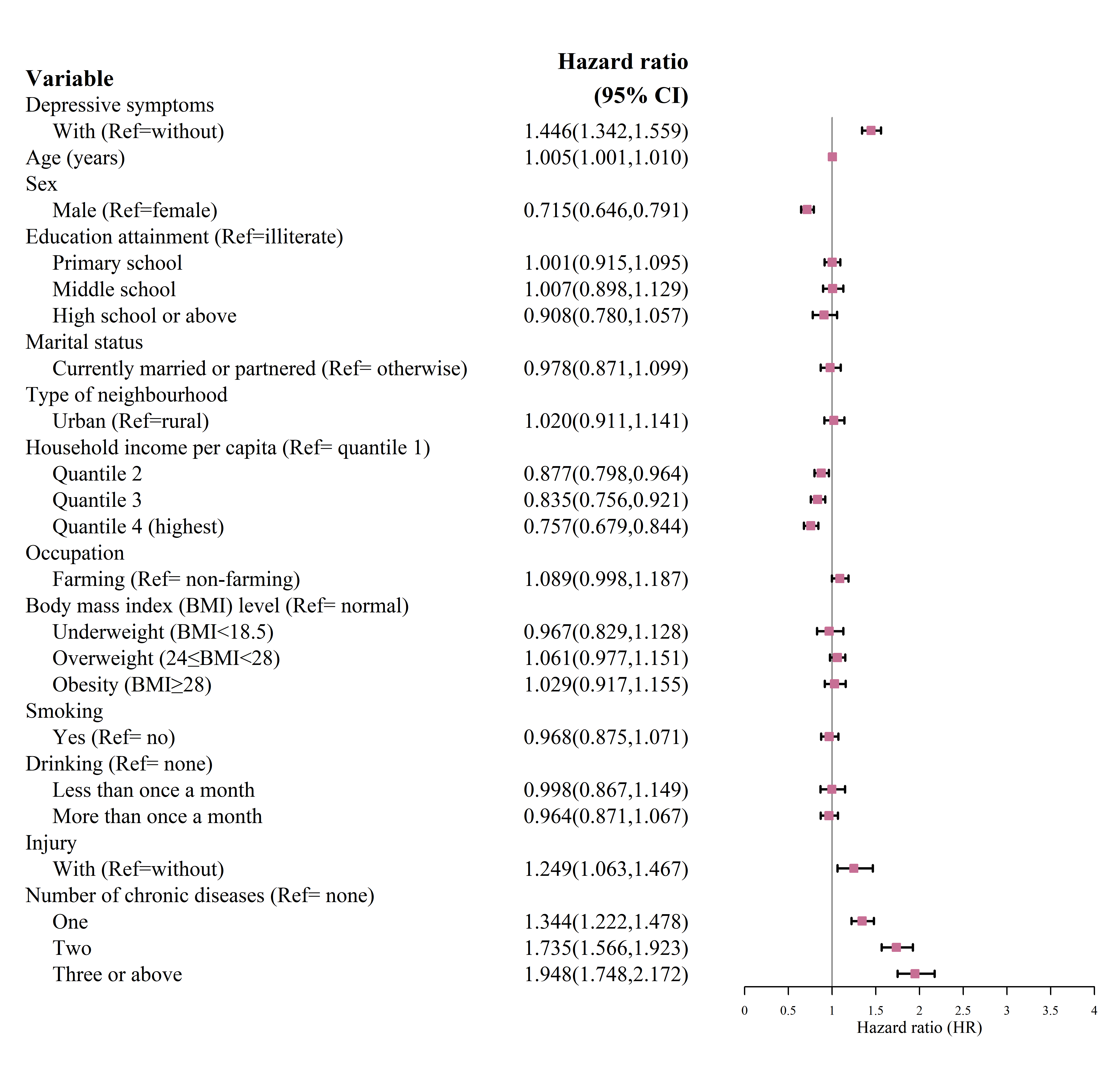

Supplement: Supplementary file 3 — Additional file 3. Association between depressive symptoms and risk of incident knee pain [file 12891_2021_4511_MOESM3_ESM.tiff]

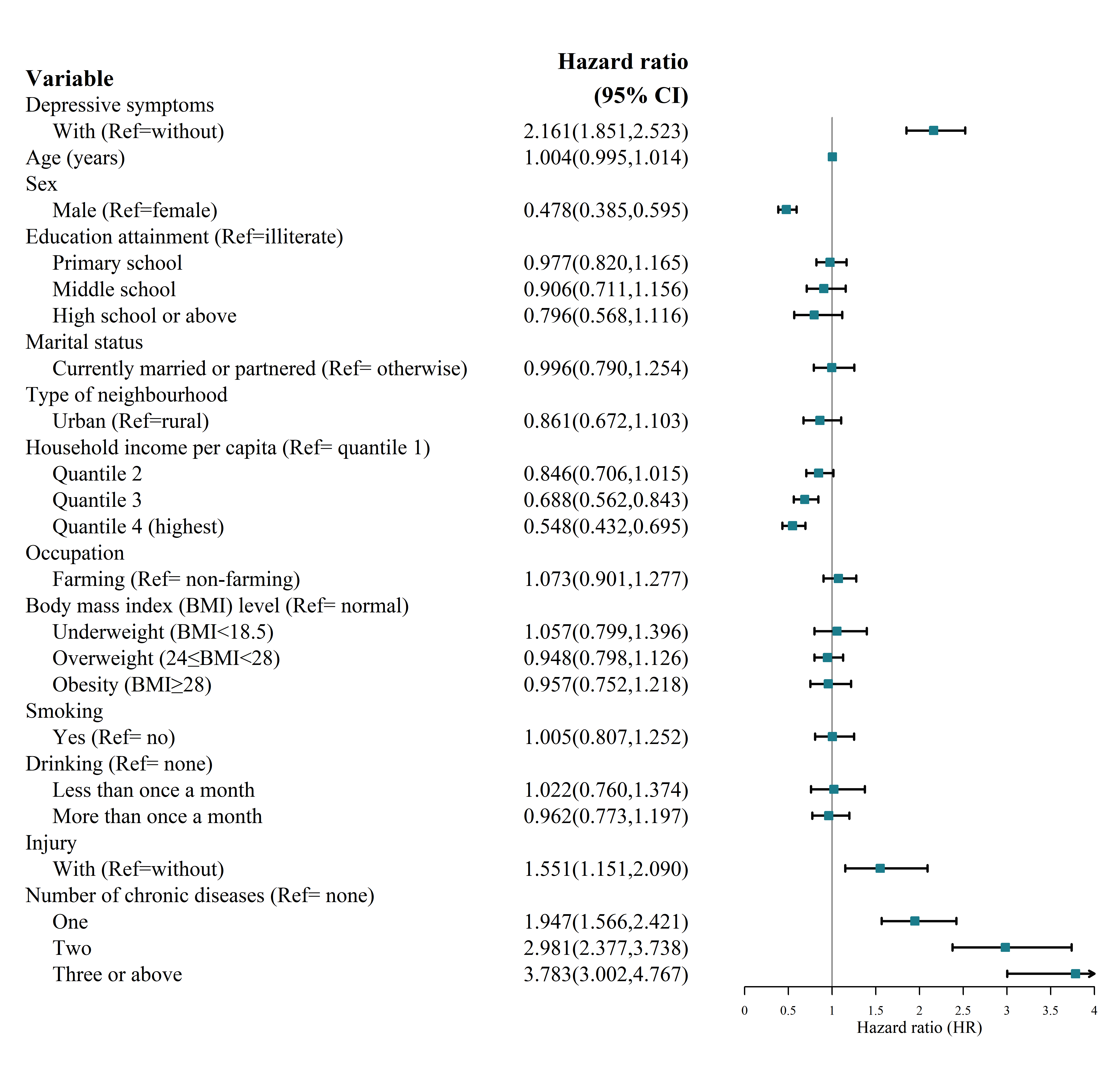

Supplement: Supplementary file 4 — Additional file 4. Association between depressive symptoms and risk of persistent knee pain [file 12891_2021_4511_MOESM4_ESM.tiff]
